# Neuromuscular Adjustments of the Quadriceps Muscle after Repeated Cycling Sprints

**DOI:** 10.1371/journal.pone.0061793

**Published:** 2013-05-01

**Authors:** Olivier Girard, David J. Bishop, Sébastien Racinais

**Affiliations:** 1 Aspetar, Qatar Orthopaedic and Sports Medicine Hospital, Doha, Qatar; 2 Institute of Sport, Exercise and Active Living and School of Sport and Exercise Science, Melbourne, Australia; University of Las Palmas de Gran Canaria, Spain

## Abstract

**Purpose:**

This study investigated the supraspinal processes of fatigue of the quadriceps muscle in response to repeated cycling sprints.

**Methods:**

Twelve active individuals performed 10 × 6-s “all-out” sprints on a cycle ergometer (recovery  =  30 s), followed 6 min later by 5 × 6-s sprints (recovery  =  30 s). Transcranial magnetic and electrical femoral nerve stimulations during brief (5-s) and sustained (30-s) isometric contractions of the knee extensors were performed before and 3 min post-exercise.

**Results:**

Maximal strength of the knee extensors decreased during brief and sustained contractions (∼11% and 9%, respectively; P<0.001). Peripheral and cortical voluntary activation, motor evoked potential amplitude and silent period duration responses measured during briefs contractions were unaltered (P>0.05). While cortical voluntary activation declined (P<0.01) during the sustained maximal contraction in both test sessions, larger reductions occurred (P<0.05) after exercise. Lastly, resting twitch amplitude in response to both femoral nerve and cortical stimulations was largely (> 40%) reduced (P<0.001) following exercise.

**Conclusion:**

The capacity of the motor cortex to optimally drive the knee extensors following a repeated-sprint test was shown in sustained, but not brief, maximal isometric contractions. Additionally, peripheral factors were largely involved in the exercise-induced impairment in neuromuscular function, while corticospinal excitability was well-preserved.

## Introduction

Most previous explanations of fatigue during repeated-sprint exercise have focused on muscular factors associated with cellular mechanisms [1]. These include limitations in anaerobic energy supply from ATP and phosphocreatine and the intramuscular accumulation of selected metabolic by-products including inorganic phosphate and hydrogen ions [2], [3]. Fatigue induced by repeated-sprint efforts has also been related to neural factors, as demonstrated by reduced muscle activation levels inferred from muscle functional magnetic resonance imaging [4], surface EMG [5], [6] or peripheral motor nerve (PMN) stimulation (*i*.*e*. twitch interpolation; [7]) techniques. In one such study, the ability to repeat ten 6-s sprints, interspersed with 30 s of rest, was associated with the occurrence of central fatigue as suggested by a significant decrement in the *vastus lateralis* root mean square (RMS) activity during the acceleration phase of each sprint [7]. Although a decrease in raw EMG activity across repetitions could be due to peripheral alterations in the transmission and propagation of the EMG signal, this study reported a significant decrement in both the normalized EMG activity (RMS/M-wave ratio) and the percentage of voluntary activation (VA) during a subsequent maximal isometric voluntary contraction [7]. Taken together, these observations suggest that the decrement in EMG induced by repeated sprints was partly linked to a central deficit in muscle activation. This was further evidenced by a significant decrease in both the percentage of peripheral voluntary activation (VA) and normalized EMG activity (RMS/M-wave ratio) during a maximal isometric voluntary contraction of the knee extensors performed within 5 min of the last sprint bout [7]. A limitation of these conventional methods however, is that the precise locus of the impaired neural drive to the muscle, which can theoretically be mediated at any site proximal to the motor axons, cannot be ascertained [8].

Transcranial magnetic stimulation (TMS) of the motor cortex, in conjuncture with the “classical” twitch interpolation methodology, has the ability to establish whether voluntary output from the motor cortex is insufficient to drive the motor neuron pool optimally [9]. Using this technique, the role of supraspinal fatigue on decrements in force production and VA of the quadriceps muscle has been demonstrated following exhaustive locomotor exercise [10], [11], [12]. In addition, EMG responses to TMS, which under fatigue may experience a change [13], [14], provide information on the dynamics of the excitability of corticospinal and intracortical facilitatory/inhibitory pathways (*i*.*e*. motor evoked potential amplitude and silent period duration, respectively). At present, the nature of the neural adjustments and the extent to which corticospinal responsiveness is altered after the completion of repeated-sprint exercise are yet to be elucidated.

The aim of this study was to provide a comprehensive assessment of the changes in cortical VA and peripheral neuromuscular function of the quadriceps muscle group in response to a repeated-sprint cycling exercise by using, for the first time, motor cortex stimulations during brief (5-s) and sustained (30-s) maximal isometric voluntary contraction (MVC). It was hypothesized that, in addition to the large peripheral fatigue, supraspinal factors would contribute substantially to exercise-induced neuromuscular adjustments.

## Methods

### Ethical statement

This study conformed to the standards sets by the latest revision of the Declaration of Helsinki and was approved by the Research Committee and the Ethics Board from Aspetar, Qatar Orthopaedic and Sports Medicine Hospital in Doha, Qatar. All subjects gave written informed consent prior to the commencement of the study, once the experimental procedures, associated risks, and potential benefits of participation had been explained.

### Subjects

Twelve active males (mean ± S.D; age 30.6 ± 4.6 years, body mass 78.8 ± 7.5 kg, stature 178.9 ± 5.7 cm) participated in this study. All subjects were training 2–3 times a week (average of 5.3 ± 4.1 h.wk^−1^) for the 6 months preceding the experiments and incorporated aerobic, repeated sprints, plyometric and/or resistance exercises into their training routines. They were asked to avoid vigorous exercise and alcohol for 24 h, caffeine for 12 h and food for 2 h before the experimental trial.

### Experimental protocol

Subjects reported to the laboratory on two occasions two to three days apart, once for a familiarization session and once for an experimental session involving the completion of neuromuscular tests before (pre-tests) and after (post-tests) a repeated-sprint exercise.

#### Familiarization visit

During the first visit subjects were accustomed with the testing procedures (*i*.*e*. habituation of the electrical stimulation to the femoral nerve and TMS of the motor cortex) used to assess muscle function. Each subject first carried out a warm-up, which consisted of ten isometric knee extensions (alternating 4 s of contraction and 6 s of rest). Subjects were instructed to progressively increase contraction intensity in order to reach the maximal levels during the last three trials. They were then requested to perform brief, submaximal and maximal MVCs of the knee extensors until they felt accustomed with the equipment (*i*.*e*. coefficient of variation in three successive MVC trials lower than 5%). Optimal levels of stimulation intensities to the motor cortex and femoral nerve were then determined (see below), and these levels remained constant during the rest of the protocol. They also performed the complete neuromuscular function test procedure. Thereafter, participants performed three single, maximal, 6-s sprints on a cycle ergometer (Excalibur Sport, Lode, Groningen, The Netherlands). The best performance of the three sprints was recorded and 95% of this value was used as the criterion score during the first sprint of all subsequent repeated-sprints.

#### Experimental session

**Figure 1:**
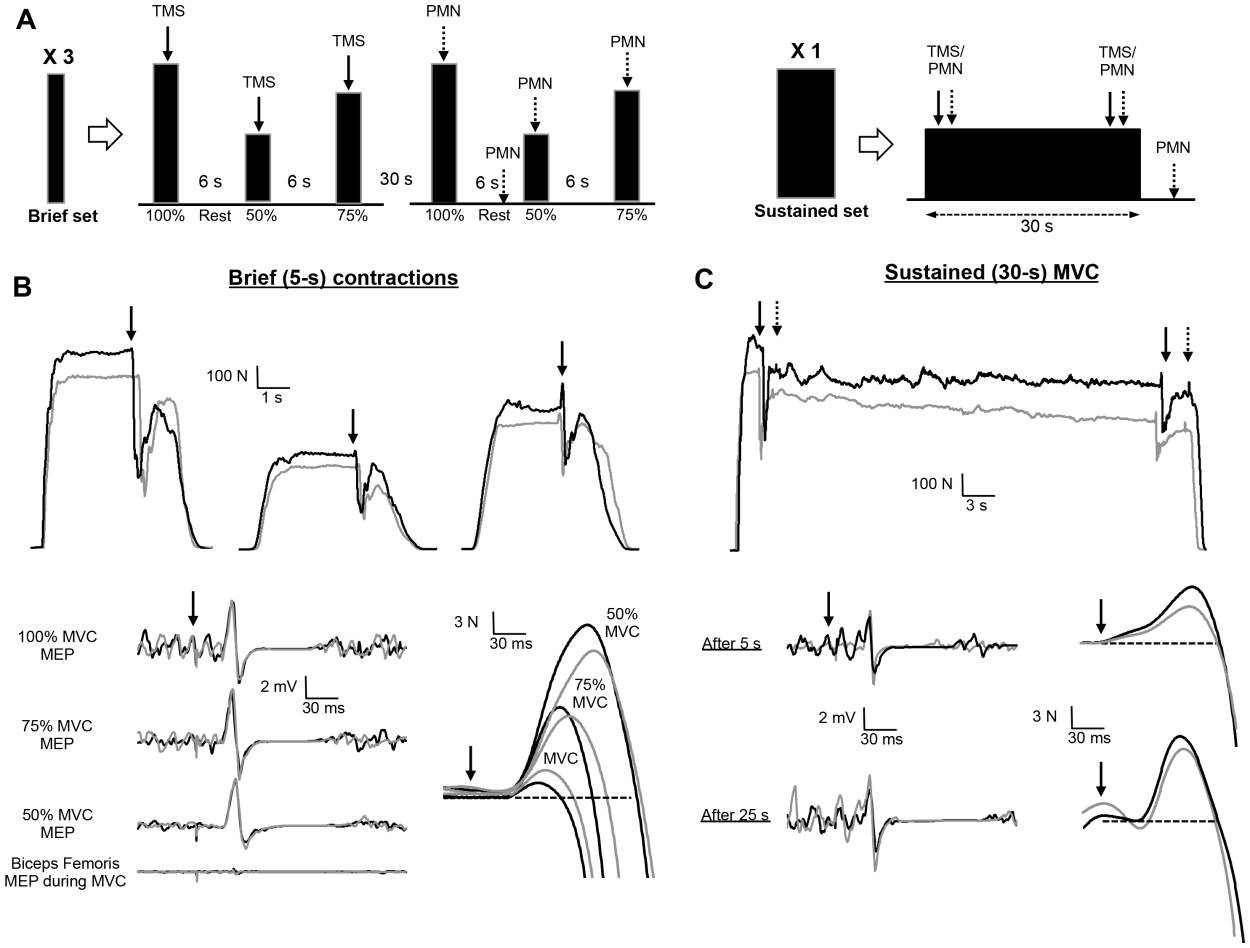
Schematic description of the neuromuscular assessment procedure which included six sets of brief (∼5 s) voluntary efforts and a sustained (30-s) maximal isometric voluntary contraction (MVC) of the knee extensors (A). Raw traces of forces and *vastus lateralis* electromyographic (EMG) responses during brief (B) and sustained (C) MVCs from a single subject before (black line) and 3 min after (grey line) the repeated-sprint test. Straight arrows indicate the timing of peripheral motor nerve (PMN) or transcranial magnetic stimulations (TMS). Each set of brief contractions involved one 100% MVC, followed by single contractions at 50% MVC and 75% MVC, during which TMS and PMN stimuli were delivered. The sustained contraction consisted of a MVC that was maintained for 30 s. The order in which TMS and PMN stimulation were applied during the brief and sustained contractions was randomized across subjects, but held constant for each person.

The experimental session was conducted as follows: (1) preparation of the subject (electrode placement, verification of the signals and stabilization of the values; ∼10 min); (2) warm-up of the knee extensors (see familiarization session); (3) a neuromuscular function (∼15 min; Figure 1) assessment (pre-tests); (4) standardized warm-up on a cycle ergometer (5 min at 75 W followed by three, sub-maximal, 6-s sprints at 70, 80 and 90% of their perceived maximum and three, all-out, 3-s sprints separated by a 90-s rest period); (5) 6 min of rest in a seated position; (6) repeated-sprint exercise (see below); (7) neuromuscular function (∼15 min) assessment (post-tests) initiated within 3 min of exercise cessation. All testing procedures were conducted in temperate ambient conditions (temperature: 24°C; relative humidity: 30%rH).

### Repeated-sprint exercise

The exercise protocol consisted of 10 × 6-s “all-out” sprints (recovery  =  30 s) on a cycle ergometer, followed 6 min later by 5 × 6-s sprints (recovery  =  30 s). Subjects were instructed to perform an “all-out” effort against a braking force (0.9 N.kg^−1^ of body mass) from the beginning of the sprint until instructed to stop. All of the subjects satisfied the 95% criteria on the first sprint. Toe clips were used to secure the feet to the pedals. Strong verbal encouragement was provided during each sprint. All of the sprints were performed from the same initial pedal position with the front pedal crank approximately 45° to the horizontal to facilitate the best starting push. During the subsequent 30-s rest period after each sprint, subjects remained seated on the ergometer. Water was provided *ad libitum* throughout the protocol.

### Neuromuscular function

Neuromuscular assessment included six sets (recovery  =  1 min) of three brief contractions (∼5 s, MVC, 50% MVC and 75% MVC, recovery  =  6 s) of the knee extensors (Figure 1). The intensities for the sub-maximal contractions were calculated from the preceding MVC, and the feedback of the target force was provided via a computer monitor. During brief contractions, TMS or PMN stimulations were alternatively delivered ∼1.5 s after the plateau (3 sets with TMS and 3 sets with PMN). In addition, a potentiated twitch was evoked 5 s after each MVC with PMN. Thereafter, subjects performed a 30-s sustained MVC including PMN and TMS, 2 s apart, delivered at the onset and just before the end of the sustained MVC (at ∼5 and ∼25 s, respectively) [12].

### EMG and force recordings

Isometric knee extensor force of the right leg was measured during both voluntary and evoked contractions on a custom-made dynamometric chair. Subjects were seated with both the hip and the knee at 100° (full extension represents 180°), one strap around the chest and one other around the hip, and the ankle tied to a strain gauge (Captels, St Mathieu de Treviers, France) connected to a stationary bench. Subject position information was recorded to ensure identical positioning for each test occasion.

Surface EMG activity of the right *vastus lateralis* and *biceps femoris* muscles was recorded using bipolar Ag/AgCl electrodes (Ambu Blue sensor T, Ambu A/S, Denmark; diameter  =  9 mm; inter-distance electrode  =  30 mm) fixed lengthwise over the muscle belly. The reference electrode was attached to the right wrist. Low impedance between the two electrodes was obtained by abrading the skin with emery paper and cleaning with alcohol. EMG signals were amplified (gain  =  1000), filtered (bandwidth frequency: 30 to 500 Hz) and recorded (sampling frequency  =  2000 Hz) by commercially-available hardware (Biopac MP35, systems Inc., Santa Barbara, CA) and its dedicated software (Acqknowledge 3.6.7, Biopac Systems Inc., Santa Barbara, CA). A 50-Hz line filter was applied to the EMG data to prevent interference from electrical sources. The root mean square (RMS) of the EMG signal was calculated over a 5-s epoch after excluding the first pedal revolution.

### Motor nerve stimulation

Single supramaximal electrical stimuli (max voltage 400 V, rectangular pulse of 200 µs) were delivered to the right femoral nerve using a high-voltage, constant-current, stimulator (Digitimer DS7AH, Welwyn Garden City, Hertfordshire, UK). The cathode ball electrode was manually pressed into the femoral triangle (*i*.*e*. 3–5 cm below the inguinal ligament) by the experimenter, and the anode (5 × 9 cm) was located in the gluteal fold opposite the cathode. The intensity of stimulation was determined at the beginning of the session by delivering single stimuli with increments of 10 mA until plateaus occurred in twitch amplitude and M-wave. Supramaximal stimulation was ensured by increasing the final intensity by 50% (mean current: 150 ± 52 mA; range 60–250 mA).

### Transcranial magnetic stimulation

A magnetic stimulator (Magstim 200, The Magstim Company, Dyfed, UK) was used to stimulate the motor cortex. A single TMS pulse (1-ms duration) was delivered via a concave double-cone coil (13 cm diameter) maintained manually over the vertex of the scalp. The coil was slightly moved to preferentially activate the left motor cortex (contralateral to the right leg) until eliciting the largest MEP in the *vastus lateralis* with only a small MEP in the *biceps femoris* (< 20% of the maximal *vastus lateralis* MEP amplitude) during 50% MVC contractions and with a stimulation intensity of 60% of the maximal stimulator power output. The optimal stimulation site (mean position: 1 cm lateral to the vertex) was marked directly on the scalp with indelible ink to ensure reproducibility of the stimulation conditions for each subject throughout the entire protocol. Afterwards, motor threshold for the *vastus lateralis* was identified for each individual by constructing a stimulus-response curve during 50% MVC contractions. Stimulator output was increased in 3% steps from 30% of stimulator output until the motor threshold, defined as the lowest stimulator intensity to elicit MEP (>50 µV) in at least three of five trials (6 s of rest between contractions). Motor threshold occurred at 41 ± 10% of maximum stimulator output, and during each of the experimental trials TMS was delivered at 140% of the motor threshold (58 ± 13% of maximum stimulator output; range: 42–87%).

### Data analysis

#### Repeated-sprint exercise

Peak power output was recorded and used to calculate the sprint decrement score (%) as follows: [1 - (total power/ideal power)] × 100 [1]. In this formula, total power is the cumulated peak power output across repetitions, while ideal power refers to the highest peak power output during one single 6-s sprint multiplied by the number of repetitions. Values for root-mean-square (RMS) of the *vastus lateralis* were determined during each sprint (Acqknowledge 3.6.7, Biopac Systems Inc., Santa Barbara, CA).

#### Neuromuscular function test

Voluntary torque and EMG activity (RMS) were recorded during 1-s of plateau before delivering TMS or PMN stimulation for all contractions (brief MVC, 50% and 75% MVCs, at the onset and at the end of the sustained MVC). Raw RMS data were also were normalized to the superimposed M-wave as an index of neural drive (*i*.*e*. RMS/M ratio).

Peripheral VA was assessed using twitch interpolation. Briefly, the force produced during a superimposed twitch during the MVC was compared with the force produced by a potentiated twitch: Peripheral VA (%)  =  (1 – [superimposed twitch/potentiated twitch]) × 100. Cortical VA was assessed by measuring the force responses to motor cortex stimulations during submaximal and maximal contractions [14]. Because corticospinal excitability increases during voluntary contraction [15] it was necessary to estimate, rather than measure directly, the amplitude of the resting twitch evoked by motor-cortex stimulation. During the sets of brief maximal and submaximal contractions (100% MVC followed by 50% and 75% MVC contractions), TMS was delivered, and the resting twitch was estimated by extrapolation of the linear relation between the amplitude of the superimposed twitch and voluntary force [16]. One regression analysis was performed for each set of brief contractions. The y-intercept was taken as the estimated amplitude of the resting twitch evoked by TMS [9], [14], [17]. The amplitude of the estimated resting twitch can be accurately determined from three data points in fresh or fatigued muscle when the contractions are > 50% MVC [9]. Data points were excluded (n = 6.9%) for subjects at different time points when the regression of the estimated twitch was R^2^<0.85. Cortical VA (%) was subsequently quantified using the equation: (1 – [superimposed twitch/estimated resting twitch]) × 100. The reliability of TMS for the assessment of cortical VA and estimated resting twitch for the knee extensors has been established elsewhere [17], [18].

Muscle contractility was assessed from the electrically-evoked resting twitch as peak twitch amplitude (*e*.*g*. the highest value of twitch tension production), time to peak twitch (*e*.*g*. the time from the origin of the twitch to the peak twitch amplitude), one half-relaxation time (*e*.*g*. the time to obtain half of the decline in maximal force), maximal rate of force development (*e*.*g*. maximal value of the first derivative of the force signal) and maximal rate of force relaxation (*e*.*g*. the lowest value of the first derivative of the force signal). The estimated resting twitch evoked by TMS was also used as an index of the force-generating capacity of the knee extensors.

The peak-to-peak amplitudes of evoked M-wave and MEP were measured offline, and the amplitude of MEP was normalized to that of the M-wave (*i*.*e*. MEP/M ratio) elicited at the same force. The duration of the cortical silent period evoked by TMS was determined as the interval from stimulus to return of continuous EMG by visual inspection [12]. The average of three (PMN- and TMS-related parameters) and or six trials (force and RMS activity data) was used for data analysis.

### Statistical analysis

Data are expressed as means ± SD (text and the tables) and means ± SE (figures). Normal distribution of the data was tested using the Kolmogorov-Smirnov test. Spericity (homogeneity of covariance) was verified by Mauchly's test. A one-way ANOVA with repeated measures (sprint number) was used to investigate modifications in peak power output and EMG-related parameters during the repeated-sprint exercise. Student's paired *t-test* was used to compare measurements obtained before (pre-tests) and after (post-tests) the repeated-sprint test. For the sustained trial, a two-way ANOVA for repeated measures [Time (pre-tests *vs*. post-tests) × Contraction duration (onset *vs*. end)] was used. Multiple comparisons were made with the Tukey HSD *post hoc* test when the Greenhouse-Geisser epsilon correction factor was P>0.05, or with the Bonferroni post hoc test when the epsilon was P<0.05. For each ANOVA, partial eta-squared (η^2^) was calculated as measures of effect size (ES). Values of 0.01, 0.06 and above 0.14 were considered as small, medium and large, respectively. Statistical analyses were undertaken by using the SPSS statistical package (version 18.0, SPSS, Chicago, Illinois).

## Results

### Repeated-sprint exercise

**Figure 2:**
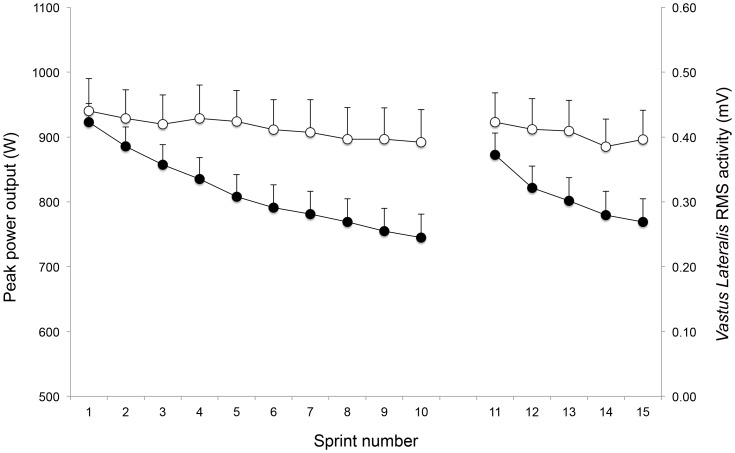
Peak power output and *vastus lateralis* Root Mean Square (RMS) EMG activity during the 15 sprints. Data are presented as means ± SE (*N* = 12). Note that there was a main effect of time (both P<0.001) for peak power output and RMS EMG activity.

During the repeated-sprint exercise there was a significant decrease (P<0.001; η^2^ = 0.81) in peak power output across the sprint repetitions (Figure 2-A). During the last 5 sprints (11–15), peak power output decreased (P<0.001) by 12.6%. The 1–10 and 11–15 sprint decrement scores were 12.0±4.8% and 7.4±4.8%, respectively. Concomitantly, statistical analysis revealed a global decrease in raw RMS activity (P<0.001; η^2^ = 0.25) (Figure 2-B). There was a strong linear relationship (r = 0.89; P<0.001) between the changes in peak power output and EMG RMS from the *vastus lateralis* muscle during the 15 sprints.

### MVC force

**Figure 3:**
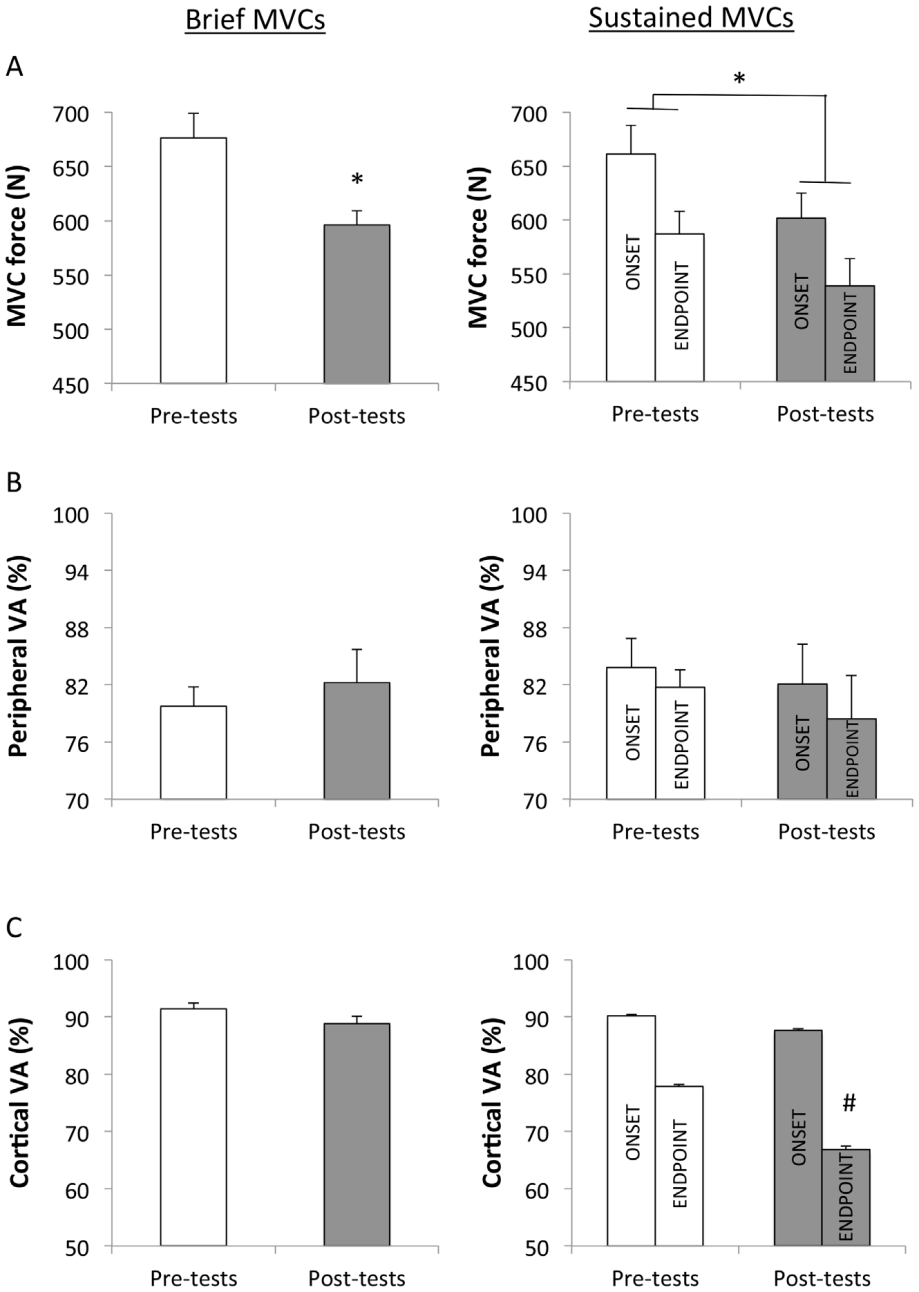
Voluntary force (A), peripheral (B) and cortical (C) voluntary activation during brief (5-s; left panels) and sustained (30-s; right panels) maximal isometric voluntary contractions before (pre-tests) and after (post-tests) the repeated-sprint exercise (mean ± SE, n = 12). During the 30-s sustained MVC measurements were obtained at the onset (∼5) and at the endpoint (∼25 s) of the contraction. * Significantly different from pre-tests (P<0.05). # Irrespectively of time, different from the onset of the contraction (P<0.05). Note that there was a significant interaction (P<0.05) between time and contraction duration for cortical voluntary activation.

Following exercise, MVC force decreased (−11.4%; P<0.001; η^2^ = 0.64) significantly from 676±103 to 596±98 N. Force produced during the sustained 30-s MVC was reduced from pre- to post-exercise (−8.6% and −8.3% after 5 s and 25 s, respectively; P<0.001; η^2^ = 0.67) (Figure 3-A). Despite this main effect of time, the extent of decline in voluntary force from the onset to the end of the sustained effort was similar before *versus* after the repeated-sprint exercise (−10.4% *versus* −10.2%; P<0.004; η^2^ = 0.54).

### Voluntary activation

During brief MVCs, peripheral (P = 0.357; η^2^ = 0.08) and cortical (P = 0.146; η^2^ = 0.18) VA were not significantly different before and after the repeated-sprint exercise (Figure 3-B,C). There was no significant main effect of time (P>0.05; η^2^ = 0.08), contraction duration (P>0.05; η^2^ = 0.12) or any interaction between these two factors (P>0.05; η^2^ = 0.02) for the peripheral VA measured during the sustained MVC (Figure 3-B). Mean cortical VA produced during the sustained 30-s MVC tented to be reduced (P = 0.085; η^2^ = 0.25) with time (−2.7% and −13.6% after 5 s and 25 s, respectively) (Figure 3-C). There was a significant interaction between time and contraction duration (P<0.05; η^2^ = 0.34), and the rate of decline in cortical VA during the sustained 30-s contraction was larger after compared to before (−24.3% *vs*. −13.4% from the onset to the end of the sustained MVC, respectively) the repeated-sprint exercise.

### EMG activity

RMS EMG activity (both raw and normalized values) and M-wave peak-to-peak amplitudes measured at rest (8.1±4.1 *vs*. 6.5±3.4 mV; P = 0.201; η^2^ = 0.16) or in any other condition did not change in response to the fatigue protocol (Tables 1 and 2).

**Table 1:** EMG-related parameters obtained during brief (5-s) voluntary contractions before (pre-tests) and after (post-tests) the repeated-sprint cycling exercise.

Variables	50% MVC		75% MVC		100% MVC	
	Pre-tests	Post-tests	Pre-tests	Post-tests	Pre-tests	Post-tests
Raw RMS (µV)	94 ± 56	84 ± 60	158 ± 93	154 ± 100	227 ± 117	209 ± 117
M-wave amplitude (mV)	9.1 ± 4.3	8.4 ± 4.2	9.4 ± 4.2	8.4 ± 4.0	10.3 ± 4.5	8.6 ± 4.3
MEP amplitude (mV)	3.6 ± 1.6	3.2 ± 1.8	4.3 ± 1.9	4.1 ± 2.4	4.2 ± 1.8	3.5 ± 2.2
Silent period duration (ms)	112 ± 17	116 ± 16	103 ± 15	108 ± 13	97 ± 8	99 ± 8
Ratio RMS/M (a.u)	0.009±0.004	0.010±0.006	0.015±0.005	0.018±0.007	0.022±0.006	0.025±0.007
MEP amplitude (%Mwave)	41 ± 16	44 ± 23	49 ± 17	49 ± 12	42 ± 9	40 ± 10

Data are mean ± SD for 12 subjects. Note that there was no time effect for any variable.

**Table 2:** Neuromuscular parameters obtained at the onset and at the end (∼5 and 25 s) of the sustained (30-s) maximal voluntary contraction before (pre-tests) and after (post-tests) the repeated-sprint cycling exercise.

		After 5 s				After 25 s		
		Pre-tests		Post-tests		Pre-tests		Post-tests
Raw RMS (µV)		212 ± 103		199 ± 112		224 ± 120		200 ± 116
M-wave amplitude (mV)		9.8 ± 5.4		6.5 ± 4.1		12.0 ± 4.1		7.2 ± 4.4*
MEP amplitude (mV)		3.9 ± 2.0		3.3 ± 1.9		4.2 ± 1.7		3.9 ± 2.0 #
Silent period duration (ms)		100 ± 11		96 ± 9		129 ± 23		118 ± 20 * #
Ratio RMS/M (a.u)		0.059 ± 0.027		0.067 ± 0.029		0.056 ± 0.028		0.054 ± 0.023
MEP amplitude (%M-wave)		40 ± 16		40 ± 14		53 ± 30		61 ± 33 *
TMS superimposed twitch amplitude (N)		18 ± 13		11 ± 11		32 ± 20		31 ± 19 #

Data are mean ± SD for 12 subjects.; RMS, Root Mean Square; M-wave, muscle compound action potential; MEP, motor evoked potential.

* Irrespectively of the contraction duration, different from pre-tests (P<0.05).

#Irrespectively of time, different from the onset of the contraction (P<0.05).

### MEPs and silent period

During brief contractions, MEP/M ratios and MEP silent periods were not affected by time at any contraction strength (Table 1). The silent period duration lengthened from the onset to the end of the sustained MVC (P<0.05; η^2^ = 0.35), while no significant modification was observed for the MEP/M ratio (Table 2). The repeated-sprint exercise increased the MEP/M ratio (P<0.05; η^2^ = 0.33) and shortened the MEP silent period duration (P<0.05; η^2^ = 0.35) measured during the sustained 30-s MVC, independently of the contraction duration (Table 2).

### Peripheral fatigue

**Figure 4:**
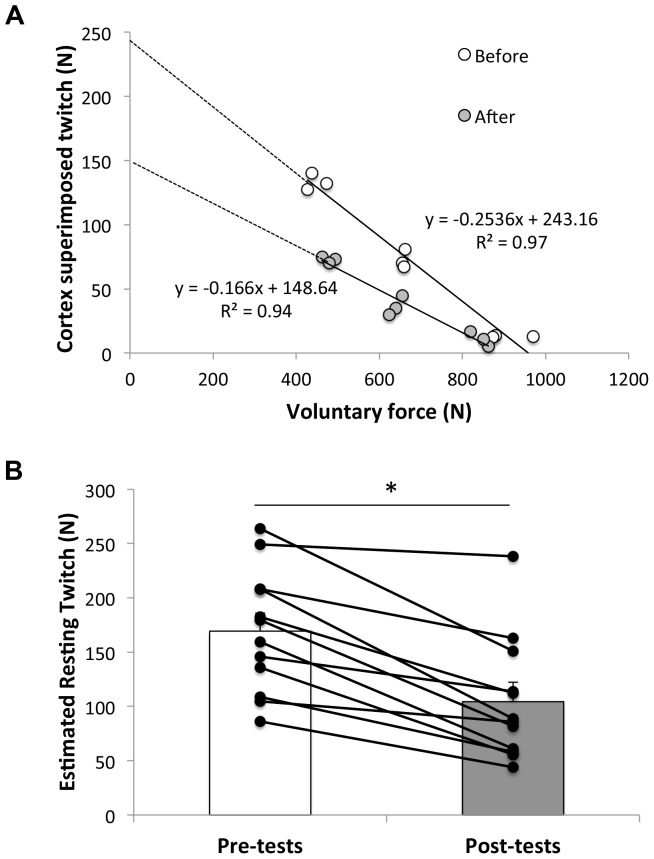
An example of the regression lines used to estimate the twitch evoked by TMS in resting muscle state from a single subject (A), and individuals and population average estimated resting twitch (B) before and after the repeated sprint exercise (mean ± SE, n = 12). * significantly different from pre-tests (P<0.05).

From pre- to post-exercise, the twitch evoked by PMN was characterized by lower peak torque (167±26 *vs*. 95±07 N; -43.2%; P<0.001; η^2^ = 0.93), shorter half-relaxation (82.2±4.5 *vs*. 74.1±4.1 ms; −9.5%; P<0.01; η^2^ = 0.53), but not contraction (67.6±5.8 *vs*. 70.7±10.1 ms; +4.8%; P = 0.295; η^2^ = 0.10) times together with slower maximal rates of force development (4.60±0.30 *vs*. 2.41±0.20 Nm.ms^−1^; −47.0%; P<0.001; η^2^ = 0.93) and relaxation (2.19±0.60 *vs*. 1.16±0.30 Nm.ms^−1^; −44.7%; P<0.001; η^2^ = 0.90). Compared to pre-tests, the potentiated twitch obtained after the 30-s MVC was lower during post-tests (164±25 *vs*. 109±25 N; −32.8%; P<0.001; η^2^ = 0.87). The estimated resting twitch from TMS decreased by 39.7% from pre- to post tests (169±55 *vs*. 104±59 N; P<0.001; η^2^ = 0.77) (Figure 4). The rate of muscle relaxation measured during the silent period of brief MVCs increased with time (+20.9%; P = 0.05; η^2^ = 0.30) from 13.5±4.9 to 15.7±4.3 s^−1^. There was a main effect of time (P<0.05; η^2^ = 0.44), contraction duration (P<0.001; η^2^ = 0.85) and an interaction between these two factors (P<0.001; η^2^ = 0.64) for the rate of muscle relaxation measured during the sustained MVC (Table 2).

## Discussion

This study is the first to investigate the supraspinal processes of fatigue of the quadriceps muscles in response to a repeated-sprint cycling exercise. The novel findings provided by motor cortex stimulations are that i) exercise-induced reductions in cortical voluntary activation were seen in sustained, but not brief, maximal isometric contractions of the knee extensors and ii) our repeated sprints cycle protocol did not impair the corticospinal responsiveness of motor neurons. In addition, our results confirmed that quadriceps fatigue following repeated all-out sprints is mainly of peripheral original.

### Neuromuscular activity during sprinting

Although a variety of repeated-sprint exercises exist, the current protocol was chosen as it has been shown to induce substantial levels of mechanical output impairments [5]. Previous studies have reported a concurrent decline in mechanical performance and the amplitude of EMG signals across sprint repetitions when the fatigue level is substantial (*i*.*e*. sprint decrement score >8% as observed here from sprint 1 to 10) [5], [6], [7]. In our study, changes in quadriceps RMS activity were positively (r = 0.89; P<0.001) associated with the changes in mechanical output. However, a reduction of RMS does not necessarily imply “central fatigue”, and to further test the hypothesis that there may have been a failure to fully activate the contracting musculature we determined muscle activation levels *via* twitch interpolation.

#### Muscle activation

During both brief and sustained MVCs, twitch-interpolation and EMG results showed that the ability to drive the muscle maximally during isolated isometric contractions of the knee extensors was not significantly affected by the completion of the present repeated-sprint exercise. Although using single twitches instead of tetani may have underestimated the maximal voluntary capacity [19], the method using single stimulus is reliable and adequate for estimating voluntary drive [20]. This stimulation procedure has been used successfully to detect a central activation failure (*i*.*e*., the voluntary activation level decreased by ∼3% from pre to post-exercise) following ten 6-s sprints interspaced by 30 s of recovery [7], while also causing less discomfort for the subject. The discrepancy between our findings and those reported by Racinais et al. [7] on peripheral VA may have resulted from differences in the subject characteristics (age, training background), or the exercise (sprints number, timing of the neuromuscular assessment) or stimulation (subject positioning on the dynamometric chair, amperage of stimulations) protocol.

The question of whether a suboptimal output from the motor cortex contributes to the impaired neuromuscular function of exercising muscles after repeated-sprint exercise has been considered previously. In one study, the absence of change in grip strength and associated EMG activity of the *flexor digitorum* muscle - *i*.*e*. a muscle group not involved in the repeated-sprint running exercise - suggested no evidence of supraspinal component to impaired performance [21]. For the first time following repeated-sprint exercise, however, we have applied motor cortex stimulations during MVCs to directly assess the completeness of quadriceps cortical VA.

Of importance, cortical VA values, determined during brief MVCs, or at the commencement of the sustained MVC, were comparable between all efforts, and were in the range (*i*.*e*. 90–95%) of those obtained in previous investigations focusing on the knee extensors [11], [12], [22]. An interesting finding of this study is that repeated sprinting did not affect the capacity of the motor cortex to maximally drive knee extensor muscles during maximal efforts lasting less than 5 s. During 30-s maximal voluntary efforts, however, subjects were unable to sustain the same corticospinal output to maximally activate motoneurons, and this effect was larger after the repeated-sprint exercise despite a similar time-dependent decrease in sustained force. Recently, Sidhu et al. [12] demonstrated that supraspinal fatigue also played a greater role in sustained MVC reduction after a locomotor exercise consisting of eight, 5-min bouts of cycling at 80% of maximal workload. As a whole, this further emphasize that, in addition to a conventional brief MVC test, sustained contractions should be utilized to observe the true weakening of the cortical function that results from strenuous locomotor exercises.

The amplitude of the cortically-evoked superimposed twitch (and therefore cortical VA values) over the course of the 30-s MVC theoretically may be influenced - at least partially - by progressively impaired twitch characteristics during sustained maximal effort. Interestingly the amplitude of the twitch responses, measured during both test sessions, was similar before and immediately after the sustained MVC. These findings would indicate that a large proportion of the impairment in muscle activation with continuing maximal voluntary efforts after repeated sprinting occurs at or above the level of motor cortical output, independent of the altered twitch characteristics attributable to exercise.

### Corticospinal responsiveness

When TMS is used to activate cortical neurons during a voluntary contraction, both excitatory and inhibitory responses (*i*.*e*. as inferred from MEP amplitude and cortical silent period duration, respectively) can be recorded in the EMG [23]. During the course of a sustained MVC the MEP amplitude typically increases in size, demonstrating enhanced cortical drive to progressively less responsive motoneurons [24]. This is usually associated with a silent period that grows longer, which is the sign of an increase in the effectiveness of intracortical inhibition [25]. In this study, however, the pattern of change in these EMG responses to TMS throughout the sustained MVC was similar between the two test sessions. Our results also showed that repeated sprinting, in general, did not alter MEP amplitudes and silent period durations resulting from brief voluntary contractions in any condition. Taken as a whole, this suggests that repeating cycle sprints do not impair the corticospinal responsiveness of motor neurons, at least when evaluated from localized, isometric MVC of the knee extensors. Although fatigue-induced alteration in EMG responses to TMS has been observed during low-intensity sustained contraction [26] or during series of intermittent isometric MVCs [27] of the elbow, neither MEP amplitudes nor silent period durations measured from the *vastus lateralis* muscle were affected by two bouts of Wingate tests [10] or the completion of eight, 5 min bouts of cycling at 80% of maximum workload [12]. Given that EMG responses to TMS measured during brief MVCs can be restored in a few minutes upon cessation of exercise [28], we cannot exclude the possibility that the 3-min window between exercise cessation and the post-exercise neuromuscular tests may have been too long to record meaningful changes in corticospinal responsiveness that could have lead to reduced performance. For this reason, future studies should minimize the gap between exercise cessation and testing to confirm that repeated sprinting does not alter cortical excitation/inhibition pathways. Nevertheless, applying TMS over the motor cortex during the push-off pedaling phase (*i*.*e*. participants were requested to pedal for 2 min at 60 rpm and 100 W) in neutral and hot conditions without interruption after both submaximal and incremental cycling tasks, we failed to record any significant change in the size of MEPs [29].

### Peripheral fatigue

Although M-wave amplitude obtained during the sustained 30-s MVC decreased from pre- to post-exercise, unchanged M-waves were observed at rest and during brief voluntary contractions. The resting M-wave response has previously been reported to increase (+14%, [7]) or decrease (−16%, [21]) following various repeated-sprint exercises. While the moderate reproducibility of M-wave measurements after fatigue might partly explain this apparent discrepancy [30], our results place the site of peripheral perturbations beyond the sarcolemma.

After exercise, the amplitude of the resting twitch produced by stimulation of the femoral nerve was reduced by more than 40%. The magnitude of this exercise-induced reduction in twitch force is greater than previously observed (−9−15%; [7], [21]), which highlights the task-dependency of alterations. This may have partly resulted from differences in participant or stimulation characteristics (*e*.*g*. potentiated *vs*. unpotentiated stimulation(s), single twitches *vs*. doublets, different muscular groups considered) as well as the details of the repeated-sprint exercise (*e*.*g*. nature of the exercise protocol, timing of the post-exercise neuromuscular assessment) between studies. Furthermore, estimates of resting twitch derived from cortical stimulations [9], [16] showed similar post-exercise amplitude reductions than peak twitch force obtained with motor nerve stimulations, while also sharing similar absolute values. Slower twitch contraction and relaxation rates also accompanied the large decline in force output from the muscle. Relatively similar changes have been observed following a repeated-sprint running exercise (12 × 40 m with 30 s of passive recovery) [21]. Presumably an impairment in the excitation contraction coupling process, whose efficacy is closely related to intracellular calcium movements involving calcium release and re-pump from the sarcoplasmic reticulum and contractile protein sensitivity to calcium, may have played a predominant role here [31].

### Limitations

A recent debate has questioned the accuracy of the twitch interpolation technique for the assessment of voluntary activation. Although Taylor [8] suggested that motor nerve estimates of voluntary activation *do* provide a measure of drive to muscle, however, they *do not* measure descending drive to the motoneurons or take into account the non- linear input-output relationship of the motoneuron pool [32]. Furthermore, twitch interpolation has been applied to fatigued single muscle fibres showing an increase in force during the plateau of a isometric contraction, indicative of “central fatigue”, which is, however, impossible in single fibres [33]. Thus, an intracellular mechanism in the form of an increased tetanic calcium concentration, rather than “central fatigue”, may account for the increase in extra force evoked by an interpolated twitch during fatigue. Consequently, motor nerve estimates of voluntary activation may overestimate the contribution of “central fatigue” [34]. Nevertheless, in spite of the recent highlighted limitations [8], twitch interpolation is sufficient to reveal changes with physiological interventions in the knee extensors if the delay between the end of exercise and the time VA is measured on a dedicated ergometer (*i*.*e*. quadriceps chair) is minimized (ideally less than 2 min). Finally, because the demonstration of supraspinal fatigue does not eliminate the possibility of spinal contribution to neural adjustments, concomitant measurement of TMS-induced MEP and cervico-medullary MEP in the contracting muscle is necessary to determine the relative contribution of cortical and spinal mechanisms in the development of “central fatigue”.

In conclusion, this study investigated the neuromuscular and cortical responses of the quadriceps muscles to repeated cycling sprints. The novel finding provided by cortical stimulation is that the capacity of the motor cortex to optimally drive the knee extensors following exercise was shown in sustained, but not brief, maximal isometric contractions. This suggests that post-exercise neuromuscular adjustments may include a suboptimal drive from the motor cortex. Additionally, we reported that repeated cycle sprints do not impair the corticospinal responsiveness of motor neurons, at least when evaluated from localized, isometric MVC of the knee extensors. Finally, peripheral factors were largely involved in the exercise-induced impairment in neuromuscular function. It remains to explore whether or not the same is true when exercising in extreme environmental conditions.
